# From Kisiizi to Baltimore: cultivating knowledge brokers to support global innovation for community engagement in healthcare

**DOI:** 10.1186/s12992-018-0339-8

**Published:** 2018-02-09

**Authors:** Chidinma A. Ibe, Lopa Basu, Rachel Gooden, Shamsuzzoha B. Syed, Viva Dadwal, Lee R. Bone, Patti L. Ephraim, Christine M. Weston, Albert W. Wu, for the Baltimore CONNECT Project Team, J. Barron, R. T. Boonyasai, J. Gentry, M. C. Gibbons, I. Lief, Y. Lu, M. Rogers, M. Rosenblum, L. Purnell

**Affiliations:** 1 Center for Health Services & Outcomes Research, Department of Health Policy and Management, Johns Hopkins University Bloomberg School of Public Health, Baltimore, USA; 2 Johns Hopkins University School of Medicine, Division of General Internal Medicine, 2024 East Monument Street, Room 2-514, Baltimore, MD 21287 USA; 3 World Health Organization Service Delivery & Safety Department, Geneva, Switzerland; 4 Centre on Governance, University of Ottawa, Ottawa, Canada; 5 Department of Health Behavior and Society, Johns Hopkins University Bloomberg School of Public Health, Baltimore, USA; 6 Department of Epidemiology, Johns Hopkins Bloomberg School of Public Health, Baltimore, USA; 7 Welch Center for Prevention, Epidemiology, and Clinical Research, Johns Hopkins Medical Institutions, Baltimore, USA

**Keywords:** Reverse innovation, Bidirectional innovation, Knowledge brokerage, Community knowledge brokers, Community engagement

## Abstract

**Background:**

Reverse Innovation has been endorsed as a vehicle for promoting bidirectional learning and information flow between low- and middle-income countries and high-income countries, with the aim of tackling common unmet needs. One such need, which traverses international boundaries, is the development of strategies to initiate and sustain community engagement in health care delivery systems.

**Objective:**

In this commentary, we discuss the Baltimore “Community-based Organizations Neighborhood Network: Enhancing Capacity Together” Study. This randomized controlled trial evaluated whether or not a community engagement strategy, developed to address patient safety in low- and middle-income countries throughout sub-Saharan Africa, could be successfully applied to create and implement strategies that would link community-based organizations to a local health care system in Baltimore, a city in the United States. Specifically, we explore the trial’s activation of community knowledge brokers as the conduit through which community engagement, and innovation production, was achieved.

**Summary:**

Cultivating community knowledge brokers holds promise as a vehicle for advancing global innovation in the context of health care delivery systems. As such, further efforts to discern the ways in which they may promote the development and dissemination of innovations in health care systems is warranted.

**Trial registration:**

Trial Registration Number: NCT02222909. Trial Register Name: Reverse Innovation and Patient Engagement to Improve Quality of Care and Patient Outcomes (CONNECT). Date of Trial’s Registration: August 22, 2014.

## Background

There is a growing awareness of the need to transition from the prevailing model of global health delivery, focused on disease-specific interventions, to one targeted toward strengthening health systems [1]. Such a shift in focus requires mutual learning and sharing across countries [2, 3] and can be achieved, in part, by *reverse innovation*. Reverse innovation (RI), in the context of health care, has been championed as a vehicle for facilitating bidirectional learning between health systems around the world. It is defined as the flow of ideas and products from low- and middle-income countries (LMICs) to high-income countries (HICs) and occurs when a successful innovation in a LMIC is identified, adapted and deployed in a HIC to address an unmet need [2–4]. Mutual, bidirectional learning is achieved as LICs function as the incubators of innovative strategies that are subsequently taken up by their higher income counterparts [2–7]. Intrinsic to von Zedtwitz and colleagues’ delineation of the different types of reverse innovations, and their subsequent categorization of these types as falling under strong and weak reversal innovations, is the notion that global innovation is more sinuous than linear [5]. Rather than following a unidirectional trajectory, global innovation is multidimensional and bimodal such that while the locus of the innovation may shift, the core innovation remains intact [5].

Growing inquiry into the development and adoption of RI interventions, rooted in an acknowledgement of their utility for global health, necessitates two interlocking streams of inquiry: first, determining how to initiate health-related RI strategies to encourage bidirectional learning and information flow between LICs and HICs; and second, identifying how to discern the barriers and facilitators of RI in HICs, including the degree to which the origin of the innovation shapes perceived utility as a reversal innovation and, consequently, its uptake in HICs [6, 7].

Strategies to tackle these questions may lie with researchers in HICs, who can leverage existing infrastructure and connections with partners across the globe to identify promising LIC solutions, pilot them in HICs, and build the evidence base to support their use [4].

Accordingly, the goal of this commentary is to discuss how a cluster-randomized trial implemented by researchers affiliated with an academic health system in East Baltimore, whose express purpose was to test the effectiveness of a community engagement strategy developed in sub-Saharan Africa, cultivated knowledge brokers to facilitate the adoption of this community engagement approach in an HIC. We highlight the ways in which the structure of the trial itself stimulated knowledge brokerage at multiple levels. This positioned the study’s stakeholders to emerge as community knowledge brokers and placed them squarely on the pathway of global innovation flow.

### African partnerships for patient safety: framework for community engagement overview

In the mid-2000s, the member states of the World Health Organization’s (WHO) Africa Region initiated efforts to address patient safety, culminating in a formal agreement to endorse 12 action areas across the region [8]. WHO instituted the African Partnerships for Patient Safety (APPS) to encourage the bidirectional transfer of knowledge and joint efforts related to patient safety improvements between African hospitals twinned with European hospitals. The connections forged between the hospital dyads are meant to facilitate the uptake and dissemination of patient safety practices within hospitals and across health systems, working in partnership to bring changes to health delivery based on frontline realities [9].

One of the principles guiding APPS was that of community engagement, whereby relevant stakeholders (patients, personnel in partnering hospitals, and WHO APPS program staff) co-develop strategies to ensure patient safety in care delivery settings. Global partners engaged with local communities and critical stakeholders and ensured that they were involved during a partnership-driven approach to improve patient safety at the hospital level. This engagement approach was informed by evidence-based best practices for community engagement, insights gleaned from existing WHO patient programs, and patients’ experiences navigating their health systems in the context of patient safety [9]. APPS Program staff, in close collaboration with a robust network of intra-national and international healthcare workers and community partners, codified their stakeholder engagement strategy into a series of seven steps comprising the APPS Community Engagement (ACE) Approach, summarized in Table 1.

**Table 1:** APPS Community Engagement Approach

1	Establish an APPS Community Engagement Advisory Board
2	Know the Community
3	Establish an Enabling Community Engagement Environment
4	Raise Patient Quality/Safety Awareness Locally and Nationally
5	Collect Community Knowledge and Experiences
6	Ensure Robust Communication Mechanisms
7	Feed into Monitoring and Evaluation
8	Develop a Community Ripple Effect

### Knowledge brokering to facilitate the adoption of the ACE approach in Baltimore

While there is evidence to suggest that the ACE Approach has been effective in sub-Saharan Africa for galvanizing stakeholder involvement and support for patient safety initiatives within participating communities [9, 10], it is unknown whether or not the use of this framework **is** efficacious in HICs that similarly struggle with engaging multiple stakeholders in the health care delivery system. A facet of this community engagement framework that bears consideration for its uptake in HICs is its cultivation of “knowledge brokers,” who not only supported the generation, translation, and dissemination of evidence-based best practices in patient safety between twinned hospitals as a part of the program; but also, the development of the community engagement process itself [10]. Knowledge brokers are intermediary individuals, organizations, or structures that develop relationships and networks with users and producers of knowledge [11]. They strengthen relationships between program developers/researchers and end users, pursuing opportunities to promote and nurture mutually beneficial linkages between partnering entities [11–13]. Bornbaum and colleagues’ systematic review of knowledge brokers, which explored the predominant functions and effectiveness of knowledge brokers in health care settings, confirm that the roles that knowledge brokers fulfill fall under three overarching domains: knowledge managers, linkage agents, and capacity builders [13].

The Baltimore CONNECT (Community-based Organizations Neighborhood Network: Enhancing Capacity Together) study was developed to test the possibility of the ACE Approach as a reverse innovation. In accordance with the taxonomy of reversal innovations developed by von Zedtwitz et al., we classify this project broadly as a strong reverse innovation. We also note that Baltimore CONNECT’s adaptation of the ACE Approach falls along the spectrum between a developing country spillover and a double reverse innovation. On the one hand, the principles underlying the ACE Framework are rooted in community-based participatory research (CBPR) and participatory action research (PAR) approaches. As such, the ACE Framework, which stems from methodologies originating in advanced countries, were codified, implemented, and evaluated in sub-Saharan Africa, and subsequently promulgated in its present form in an advanced country. On the other hand, the emancipatory educational philosophies advanced by Paulo Freire fundamentally underpins these research approaches [14], which is suggestive of a double reverse innovation insofar as the ACE Framework’s tenets were conceptualized in the Global South, developed in HICs (in its permutation as CBPR or PAR), systematized in sub-Saharan Africa, and executed in Baltimore (Table 2).

**Table 2:** Types of Organizations in Baltimore CONNECT^a^

Type of Organization	Amount in Intervention Group	Amount in Control Group
Community Association	1	2
Faith-based Institutions	2	1
Food Security	1	1
Hispanic Resources	1	1
Housing/Shelter	1	1
Employment	1	1
Multipurpose	2	2
Reentry/Substance Abuse	1	1
Bereavement	1	0
Senior Care	0	1

^a^The classification of these organizations is based on self-identification of their purpose and the main services they provide to their clients. “Multipurpose” indicates that they are all-encompassing service providers

Regardless of the classification, from its inception in September 1, 2013, to its conclusion on June 30, 2016, we sought to explore whether or not a community engagement strategy developed in sub-Saharan Africa, whose principal focus was patient safety, can be applied in a city located in an HIC for a different phenomenon: establishing, strengthening, and sustaining linkages between local community-based organizations and a local healthcare organization, the Johns Hopkins Health System (JHHS). Baltimore CONNECT is based in East Baltimore, an area of Baltimore City characterized by concentrated poverty, crime, poor social support, low social capital, and low neighborhood cohesion [15, 16]. These factors converge to produce complex medical, social, and community/neighborhood health needs, creating an environment that hinders achieving and maintaining individual, family, and community health. Moreover, JHHS struggles with the provision of coordinated care across the care continuum, including frontline staff who may lack of awareness about appropriate, available resources to which patients can be referred.

In view of these issues, Baltimore CONNECT posits that linking the health system (whose expertise lies in tackling medical problems) to local community-based organizations (CBOs, who tend to address the constellation of social factors that fundamentally affect individual and community health) will contribute to the improved health of East Baltimore residents, especially since several CBOs have clients who receive care at affiliated JHHS practices. We tested this overarching hypothesis by recruiting a total of 22 CBOs to participate in our study, half of which were randomized to the intervention group. We employed a stratified randomization process whereby treatment allocation was constrained based on location, client population, and the types of services provided by the organization. We adapted the ACE Approach to partner with intervention CBO leaders, to co-develop and implement a set of interventions, or “toolkit” components, aimed at building organizational capacity and stronger linkages between intervention CBOs and the health system.

The resultant toolkit comprises web-based and in-person strategies geared toward supporting the bidirectional flow of information and resources among the intervention CBOs, and between these organizations and JHHS. Web-based components of the toolkit were housed in a website featuring a subscription-based service, Health*ify*, that allowed staff to search for local resources to refer their clients to; health education materials; and organizational capacity-building information about recruiting and retaining volunteers. In-person strategies consisted of meet-and-greet sessions between CBO leaders and JHHS frontline staff to increase knowledge about, and awareness of, the services provided by each of these entities; and research assistants, who volunteered at the organizations and trained CBO staff on the use of the web-based tools. Regular meetings were another key feature of the toolkit, insofar as they proved essential to strengthening relationships between members of the study team and the intervention CBO representatives.

### Multilevel community knowledge brokerage in the pathway of bidirectional information flow

Chief among the various types of knowledge brokers that were cultivated in the Baltimore CONNECT trial were community knowledge brokers, defined by Pyper as a group of individuals who are embedded in the very communities that health services or interventions endeavor to reach [17]. In this capacity, community knowledge brokers facilitate knowledge translation and management between patients and healthcare professionals. On an *interpersonal level*, Baltimore CONNECT’S adaptation of the ACE framework was achieved by leveraging the expertise found among the study’s CBO partners and advancing their role as community knowledge brokers. Their frequent contact with study team members, research assistants, JHHS frontline staff, and, importantly, their peers and staff, not only created buy-in for the broader project and its objectives, but also, stimulated multidirectional information and innovation flow between these individuals. CBO staff and leaders learned about JHHS and were directly connected to staff and programs that could support their clients’ physical health. JHHS frontline staff became aware of the multitude of CBO initiatives that aimed to mitigate the influence of social factors affecting patients’ health. The study team grew in their knowledge of the barriers and facilitators affecting CBO functioning and capacity, and the CBO leaders had direct linkages to likeminded community leaders and researchers. Thus, community knowledge brokering, achieved through co-developing and implementing the study’s suite of interventions alongside the study’s CBO partners, created the dual emergence of the multiple stakeholders affiliated with the project as producers *and* recipients of knowledge and opportunities to disseminate it. This is particularly salient for the CBO partners, who played a pivotal role in disseminating knowledge about the study, other CBOs, and the broader local health system to their employees and constituents. Further, it is analogous to the APPS program, where community engagement strategies provided an opportunity for WHO team members, hospital staff and local thought leaders to promulgate patient safety measures among their peers in partnering hospitals, producing and using knowledge generated from best-practices implemented across their respective hospitals.

Multiple stakeholders became community knowledge brokers through the constellation of processes associated with co-development. However, this would not have occurred without knowledge brokerage operating beyond individuals to occur at the *structural level*. The concept of the study – to test a community engagement strategy as a reverse innovation – led to systematic, continuous stakeholder engagement. The provision of funds allocated to support toolkit development and execution positioned the project *itself* as an intermediary that catalyzed knowledge brokerage. Indeed, interactions between all parties affiliated with the project were a function of its presence as it advanced multistakeholder involvement through co-created direct engagement opportunities.

## Conclusion

Co-development between knowledge producers and end-users is a cornerstone of the ACE Approach. The process blurs the lines between those typically construed as creating knowledge (researchers, program developers, and content experts) and those receiving, and translating, that knowledge into policies and practices (end-users). Baltimore CONNECT systematically activated community partners as community knowledge brokers, suggesting that 1) there is an inextricable link between structural knowledge brokerage and the cultivation and sustainment of community knowledge brokers; and 2) that concerted efforts to catalyze community partners and other key stakeholders as community knowledge brokers may stimulate global innovation flow between LMICs and HICs. Given that knowledge brokerage has been situated within the broader context of strategies promoting knowledge management and translation, we suggest that future work examines both the extent to which, and the contexts under which, knowledge brokerage facilitates reverse/bidirectional innovation. Moreover, further inquiry into whether or not dimensions of knowledge brokerage lie on the causal pathway between innovation generation and dissemination between developing and advanced countries is warranted.

## Abbreviations

ACE: African Partnerships for Patient Safety Community Engagement
APPS: African Partnerships in Patient Safety
CBO: Community-based Organization
CONNECT: Community-based Organizations Neighborhood Network: Enhancing Capacity Together
HIC: High-income Country
LIC: Low-income Country
LMIC: Low- and Middle-income Country
RI: Reverse Innovation
WHO: World Health Organization
